# Uniportal video-assisted thoracoscopic (VATS) subtotal parietal pleurectomy for refractory tuberculous pneumothorax: Five case-reports

**DOI:** 10.1097/MD.0000000000033082

**Published:** 2023-03-03

**Authors:** Xiaoyu Liu, Xuan Wang, Lei Shen, Bing Wang, Li Li, Xiyong Dai

**Affiliations:** a Wuhan Pulmonary Hospital, Wuhan Institute for Tuberculosis Control, Wuhan, Hubei, China.

**Keywords:** parietal pleurectomy, pneumothorax, refractory, thoracoscopic, tuberculosis, VATS

## Abstract

This study explored the safety and feasibility of uniportal video-assisted thoracoscopic (VATS) paretal pleurectomy in patients suffering from complex tuberculous pneumothorax. These cases were reported and summarized to present the experience of the authors with this procedure.

**Methods:**

The clinical data of a total of 5 patients with refractory tuberculous pneumothorax having undergone subtotal parietal pleurectomy by uniportal VATS in our institution were hereby collected from November 2021 to February 2022, and regular follow-up was conducted after surgery.

**Results:**

Parietal pleurectomy via VATS was successfully performed in all these 5 patients, among which, 4 received bullectomy at the same time, with no conversion to open surgery. Among the 4 cases of full lung expansion who were suffering from recurrent tuberculous pneumothorax, the preoperative chest drain duration ranged from 6 days to 12 days; the operation time, from 120 minutes to 165 minutes; intraoperative blood loss, from 100 mL to 200 mL; the drainage volume, from 570 mL to 2000 mL 72 hours after operation; and chest tube duration, from 5 days to 10 days. One rifampicin-resistant case had satisfactory postoperative lung expansion, but left a cavity, the operation time of which was 225 minutes; intraoperative blood loss, 300 mL; the drainage volume, 1820 mL 72 hours after operation; and chest tube duration, 40 days. The follow-up time ranged from 6 months to 9 months, and no recurrence was noted.

**Conclusion:**

Parietal pleurectomy with preservation of the top pleura via VATS is a safe and satisfactorily effective procedure for patients with refractory tuberculous pneumothorax.

## 1. Introduction

Refractory tuberculous pneumothorax has always been an intractable problem requiring surgical treatment, and its associated comorbidities make the treatment rather demanding.^[1]^ It is characterized by multiple pulmonary bulla and recurrent spontaneous pneumothorax, which is difficult to be treated through conservative treatment, such as prolonging the drainage time or even blocking up the gap on the bronchus. Bullectomy, either via the traditional open technique or by video-assisted thoracic surgery (VATS), is not suitable for such patients due to the formation of a new fistula, and poor pulmonary function caused by the risk of long-term persistent air leakage.^[2]^ Parietal pleurectomy is mainly used for pleural biopsy with an unknow cause of the pleural effusion, also surgical treatment of stubborn pleural effusion, and spontaneous pneumothorax.^[3]^ Although there are few relevant reports, the treatment of spontaneous pneumothorax with parietal pleurectomy can significantly reduce the recurrence rate of pneumothorax.^[2,4,5]^

In view of the fact that tuberculous pneumothorax often have multiple pulmonary bullae and a poor pulmonary function, jointly with the severe and acute disease symptoms as well as its easy recurrence, the majority is refractory pneumothorax.^[6,7]^ Parietal pleurectomy should be the ideal operation in theory, but there is no relevant special report at home and abroad. Herein, the detailed surgical procedure of subtotal parietal pleurectomy was described via VATS performed for 5 patients with refractory tuberculous pneumothorax to preliminarily discuss their safety, effectiveness, and applicable objects, which has been proven effective with no recurrence during follow up.

## 2. Case reports

### 2.1. Materials and methods

#### 1.2.1. Ethics approval.

Written informed consent was obtained from the patient’s legal guardian/next of kin. The present study protocol was reviewed and approved by the Ethics Committee of Wuhan Pulmonary Hospital, Ethics No:(2022)11.

#### 2.2.1. Patients.

A total of 5 cases of refractory tuberculous pneumothorax treated by VATS parietal pleurectomy in Wuhan Pulmonary Hospital from November 2021 to February 2022 were recruited in this study. All of them were males, aged from 60 to 76 years old. Data were collected from the medical records of these patients.

#### 3.2.1. Preoperative preparation.

All patients suffered from refractory tuberculous pneumothorax and underwent conservative treatment, including closed thoracic drainage and respiratory physiotherapy, to improve the basic physical condition. All patients had undergone laboratory examination, including blood and coagulation tests, electrogram, and cardiac ultrasound, to assess their tolerance to general anesthesia. CT was performed to confirm whether the patients were good candidates for parietal pleurectomy via VATS. The patients were informed of the treatment plan as well as its advantages and disadvantages, and offered their informed consent.

#### 4.2.1. Surgical technique.

**Figure 1.**
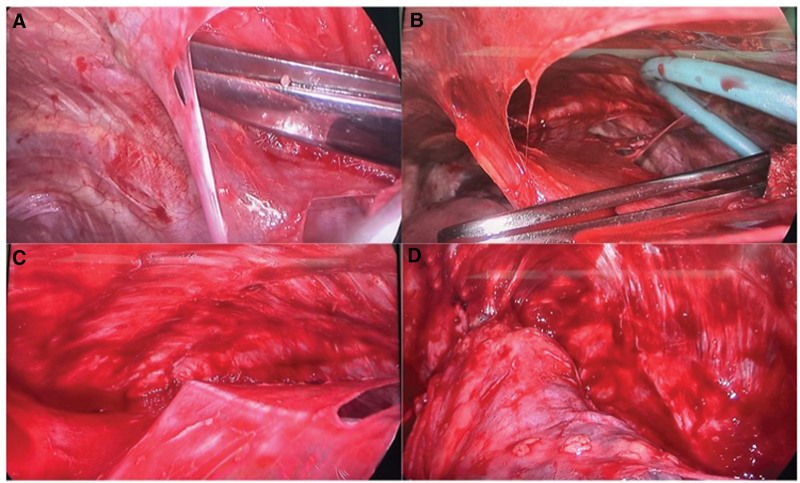
Surgical procedure.

All surgeries were carried out under general anesthesia and in the lateral decubitus position. The thoracic cavity was accessed via an incision approximately 3 cm to 4 cm long in the 4th front intercostal space at the anterior axillary line. A 1cm incision was made in the 7th intercostal space of the midaxillary line as the observation hole.^[2]^ The chest cavity was inspected after entering the thoracic cavity, pulmonary ventilation was conducted, and the large pleural fistula was marked. The parietal pleura around the incision was bluntly separated using tissue scissors through the skin incision (Fig. 1A)., and the 2 layers between the parietal pleura and visceral pleura were then continued to be bluntly separated by the blunt separator adopted by Professor Zhou research group^[2]^ (Fig. 1B–C). The parietal pleural dissection edge extended to the lower edge of the first intercostal space, down to the level of the diaphragm, anteriorly to the side of the sternum, and back to the level of the paraspinal sulcus (Fig. 1D). When the separated parietal pleura was resected, hemostasis was conducted and electrocautery was performed to control the small artery bleeding of the chest wall. Bullectomy or suture was used to treat the pleural fistula marked in front. After operation, a 28-Fr chest tube was inserted along the top of the pleura through the operation incision and 1 at the posterior costophrenic angle through the observation incision to ensure the drainage effect.

#### 5.2.1. Postoperative therapy and follow-up.

The chest radiograph was performed on the 1^st^ or 2^nd^ day after operation, the patients were encouraged to cough and get out of bed early for early activities, and measures were taken to actively deal with the comorbidity. Patients presenting restoration of the preoperative status, satisfactory lung recruitment, and no occurrence of significant complications could be discharged. Outpatient or telephone follow up was carried out at 2 weeks after discharge, and postoperative CT was conducted 6 months to 9 months after operation to evaluate the surgical outcome.

### 2.2. Results

Among 5 patients with refractory tuberculous pneumothorax, there were 3 cases of stable pulmonary tuberculosis and 2 of active pulmonary tuberculosis. These 5 patients were admitted in our hospital because of acute aggravation of chest tightness and breathlessness, and all of them were subject to comorbidities. Case 5 was in the state after gastrectomy, with aspergillus lung disease, stable COPD, and uncontrollable rifampicin resistant tuberculosis (Table 1). Cases 1, 2, 3, and 4 were treated with closed thoracic drainage ranging from 6 days to 12 days before operation, and lung nearly full expansion after the drainage. Cases 1, 2, and 4 developed subcutaneous emphysema. Case 5 did not place catheter for drainage before operation (Table 1)

**Table 1:** Demographic details.

Case	Sex	Age, yr	Serum albumin, g/L	Comorbidity	Tuberculosis	Number of pneumothorax occurrences	preoperative chest drain duration, d	Preoperative drainage effect
1	male	60	40.2	ankylosing spondylitis	stationary	2	12	nearly fully expansion, subcutaneous emphysema
2	male	75	30.1	diabetes, hypertension	active, consolidation treatment stage	2	7	nearly fully expansion, subcutaneous emphysema
3	male	73	40.2	hypertension	stationary	2	12	nearly fully expansion
4	male	76	31.2	COPD, hypertension	stationary	2	6	nearly fully expansion, subcutaneous emphysema
5	male	61	28.8	aspergillus, COPD, gastrectomy	active, rifampicin resistance, intensification treatment stage	1	0	/

**Figure 2.**
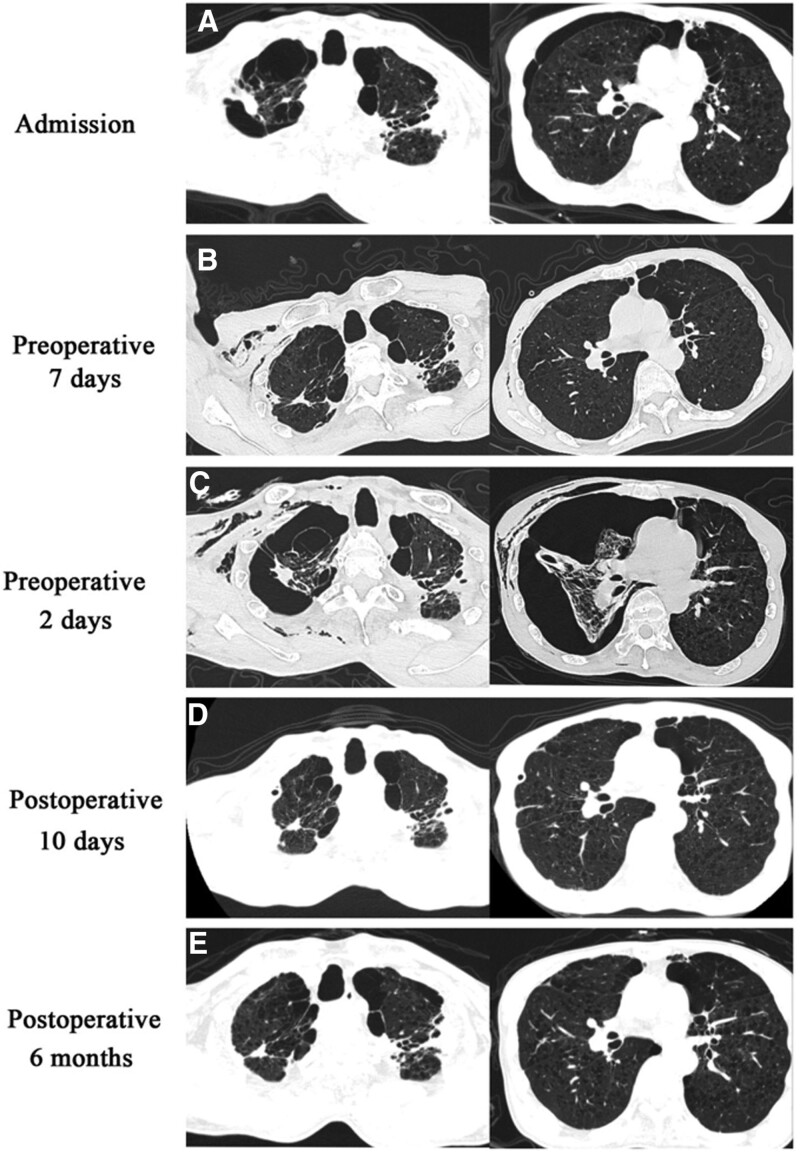
CT details of case 1. (A) On admission, (B) 7 days before operation, also the fifth day of drainage tube placement, (C) 2 days before operation, 2 days after the second drainage, (D) The 10th day after operation, and (E) 6 months after operation.

Besides Case 4 going through parietal pleurectomy by VATS, the other 4 patients underwent parietal pleurectomy and bullectomy by VATS. The operation time of Cases 1, 2, 3, and 4 was 120 to 165 minutes; the intraoperative blood loss, 100 to 200 mL; the operation time of Case 5, 225 minutes; and the intraoperative blood loss, 300 mL (Table 2). There was no secondary infection, cardio cerebral vascular accident, etc. In Case 5, due to poor appetite and low albumin, albumin was transfused for many times, and blood products were not transfused in other cases. The lungs of Cases 1, 2, 3, and 4 were completely dilated, the drainage volume was 570 to 2000 mL 72 hours after operation, and the postoperative time of catheter insertion was 5 to 10 days, shorter than the preoperative drainage time. In Case 5, the lung was reopened compared with that before operation, but there was still a cavity. The drainage volume was 1820 mL 72 hours after operation, and the tube was taken 40 days after operation. No pneumothorax recurrence/aggravation cases were found during 6 to 9 months of follow up (See Table 2 for intraoperative and postoperative details). Figure 2 shows the detailed CT changes of case 1.

**Table 2:** Intraoperative findings and postoperative recovery.

Case	Operation way	Intraoperative blood loss (mL)	Operation time (min)	Drainage volume in the first 3 days after operation (mL)	Chest tube duration time (d, 2 tubes)	Lung expansion	Respiratory improvement
1	1, 2	150	165	2000	7, 10	fully	yes
2	1, 2	200	130	800	4, 5	fully	yes
3	1, 2	200	145	1290	5, 9	fully	yes
4	3	100	120	570	4, 5	fully	yes
5	1, 2	300	225	1820	20, 40	partial	yes

Operation 1: Parietal pleurectomy (cut off all parietal pleura except the roof of pleural).

Operation 2: Bullectomy.

Operation 3: Parietal pleurectomy (resect the parietal pleura of other parts in an interval manner in addition to retaining the parietal pleura at the top of the pleura).

## 3. Discussion

The patients with tuberculous pneumothorax mostly presented acute exacerbation of chronic respiratory insufficiency, severe pleural adhesion, multiple pleural fistulas, and collateral ventilation involving multiple pulmonary segments. Multiple conditions such as proliferation, calcification, pulmonary bullae and emphysema coexisted at the same anatomical site. The drainage tube was rather easy to wrap, subcutaneous emphysema appeared, and the drainage effect was poor. The bullae could be ruptured again, which could lead to local pneumothorax. Besides, the tubes were difficult to place, and most of them were refractory pneumothorax. At present, the corresponding treatment was subject to multiple difficulties, and there were limitations in the conventional treatment of tuberculous pneumothorax. Additionally, the duration of thoracic drainage and anti-TB treatment was usually long.^[8]^ Traditional bullectomy was the only independent risk factor for long-term postoperative air leakage.^[9]^ Unlike traditional surgery, which pursued more or even complete lobectomy, parietal pleurectomy was more dependent on the formation of adhesion and closure between pleural fistula and chest wall to deal with pulmonary leakage, while the recurrence rate was reduced by removing parietal pleura.^[10]^

The 4 successful cases in this study were all decannulated within 10 days after operation. The reason why the lung reopened more quickly might be that the large fistula turned into a small fistula, thereby reducing the amount of air leakage. In addition, the subtotal parietal pleurectomy solved the problem of intrapleural separation, reduced the probability of chest tube wrapping, and ensured a better drainage effect. After operation, the chest cavity was not flushed, and the autologous blood was therefore evenly distributed in the visceral pleura, which was conducive to the rapid closure of the pleural fistula, and was also the reason for rapid lung recruitment. The reason why the patients having undergone parietal pleurodectomy were exposed to less recurrence was related to the rapid expansion of the lungs and the dense adhesion of the stripped pleural wall. This surgical method started from the pleural fistula to achieve external closure of the pleural fistula, exerting no effect on the corresponding pulmonary segment function of the fistula. No adverse events such as hemothorax were observed in these 5 cases. Considering that the operation of parietal pleurodectomy did not involve deep tissues, the incision was also minimally invasive thoracoscopic surgery. None of the 5 patients had secondary infection, and the complication rate was low, which was probably attributed to the absence of trachea during the operation.

Experience of parietal pleurodectomy in patients with tuberculous refractory pneumothorax: The possibility of lung recruitment was evaluated via preoperative drainage; Given that not all parietal pleura was resected, the term “subtotal parietal pleurectomy” was used for the procedure. The parietal pleura at the top of the pleura should be properly reserved to avoid the formation of cavities at the top of the pleura; During the operation, the large fistula could be reduced to a smaller fistula, while the treatment of pulmonary bullae could not affect the adjacent visceral pleural adhesion; The bleeding caused by pleural exfoliation should be appropriately reduced according to the situation: in Case 4, only part of the parietal pleura was separated and resected, thus leading to the minimal postoperative drainage flow, that is, septal pleurectomy could significantly reduce exudation. The incomplete re-expansion of case 5 might be attributed to the patient’s poor condition, including in the state after gastrectomy, aspergillus lung disease, stable COPD, uncontrollable rifampicin resistant tuberculosis, weak cough after surgery, no closed drainage before surgery, and insufficient evaluation of the possibility of postoperative re-expansion before surgery.

These cases suggest that video-assisted thoracoscopic parietal pleurodectomy is a safe and effective technique for the treatment of refractory tuberculous pneumothorax, applicable to cases featuring stable tuberculosis, tolerance to surgery, multiple bullae, repeated pneumothorax, long expected closed thoracic drainage time, and evaluation of lung recruitment. However, pleural atresia reduces the recurrence rate, but it is more difficult to perform lung surgery again. To this end, this operation should be carefully selected for young people or patients who need lung transplantation later. Considering the seasonal incidence of tuberculous pneumothorax, there are fewer cases in this study. Thus, more cases will be included in the next few years and the follow-up time will be extended to further verify the hereby concluded findings.

## Author contributions

**Conceptualization:** Xiaoyu Liu.

**Data curation:** Xuan Wang, Bing Wang.

**Formal analysis:** Xiaoyu Liu, Xuan Wang.

**Investigation:** Xiaoyu Liu, Xuan Wang, Lei Shen, Bing Wang.

**Methodology:** Xiaoyu Liu, Li Li, Xiyong Dai.

**Project administration:** Xiyong Dai.

**Supervision:** Li Li, Xiyong Dai.

**Validation:** Xiaoyu Liu.

**Visualization:** Xiaoyu Liu.

**Writing – original draft:** Xuan Wang.

**Writing – review & editing:** Xiaoyu Liu, Li Li.
